# Reducing the risk of bleeding after myomectomy: is preemptive embolization a valuable tool?

**DOI:** 10.1186/s42155-021-00231-9

**Published:** 2021-05-20

**Authors:** Ylann Abrahami, Sophia Najid, Arthur Petit, Eric Sauvanet, Luigi Novelli

**Affiliations:** 1 Department of Gynecologic Surgery and Obstetrics, Hopital Paris Saint-Joseph, 185 rue Raymond Losserand, 75014 Paris, France; 2 Department of Interventional Radiology, Hopital Paris Saint-Joseph, Paris, Ile-de-France France

**Keywords:** Uterine fibroids, Preemptive, Uterine artery embolization, Myomectomy, Gelatin sponge-like particles

## Abstract

**Purpose:**

Abdominal myomectomy can be a challenging procedure, with elevated intraoperative blood loss and post-operative complications such as the need for blood transfusion and hemostasis with sometimes hysterectomy. Previous studies suggested that preemptive uterine artery embolization (PUAE) might reduce intraoperative blood loss.

**Materials and methods:**

We reviewed all cases of abdominal myomectomy in our institution between January 2016 and June 2018. Out of 119 cases, 16 patients had PUAE and 103 did not. The objective of our study was to determine whereas PUAE reduced blood loss and post-operative complication rate.

**Results:**

In our study, there was no difference between the two groups in regard to average blood loss (128 vs 192 mL, OR 1,00 [0.99;1,01], *p* = 0,73), difference between pre- and post-operative hemoglobin level (− 1,15 g/dL vs − 1,32 g/dL, OR 0,91 [0.47;1,73], *p* = 0,79), and post-operative complications (need for transfusion, surgical revision, post-operative embolization, hysterectomy).

**Conclusion:**

Our findings could not conclude that PUAE is effective in reducing intraoperative blood loss during abdominal myomectomy, but it should still be considered an option for patients with large or multiple myomas, with a specific situation or previously operated, who wish to preserve their uterus.

## Introduction

Uterine leiomyoma are the most common tumors of the female genital tract, with an incidence estimated between 40 and 60% at age 35 and between 60 and 70% at age 50 in the general population. Patients with uterine fibroids are most commonly asymptomatic. Nevertheless, 20–50% of them experience symptoms such as acute pelvic pain, vaginal bleeding or infertility, and will require treatment (Vercellini et al. 1993).

Symptomatic uterine fibroid standard treatment is either conservative (hysteroscopic, laparoscopic, or open surgery myomectomy) or radical (hysterectomy) depending mainly on the patient’s childbearing desire.

These tumors are densely vascularized. Thus, myomectomy can often be a challenging procedure with a significant risk of per- and post-operative bleeding, prolonged surgical procedure duration, post-operative complications and need for transfusion. In the literature, transfusion is shown to be necessary in up to 20% of cases following abdominal myomectomy (Wallach et al. 1981; Wallach 1992; LaMorte et al. 1993).

Some fibroid characteristics have already been identified as risk factors for such complications in open surgery myomectomy: past myomectomy history, uterine volume larger than 20 weeks of amenorrhea, per-operative extraction of more than 10 fibroids, and midline incisions (Kikelomo et al. 2017).

In order to diminish the rate and severity of such complications, new medical, surgical, and combined interventional strategies have emerged and shown to significantly reduce per-operative bleeding (Kongnyuy and Wiysonge 2014).

Uterine arteries embolization (UAE) efficiency is well documented in the treatment of uterine fibroids, alone or combined with surgical myomectomy (Marshburn et al. 2006; Goodwin et al. 2008; Lohle et al. 2008; Yoon et al. 2018).

A few studies on Preemptive Uterine Artery Embolization (PUAE – maximum 24 h prior to surgery) have shown encouraging results regarding blood loss, need for per- or post-operative transfusion, surgical revision, and hemostasis hysterectomy (Schnapauff et al. 2018; Djelmis et al. 2001; Paxton et al. 2006; Üstünsöz et al. 2007; Dumousset et al. 2008; McLucas and Voorhees 2015). In 2011, Butori et al. suggested the use of gelatin sponge like particles for UAE prior to myomectomy in the treatment of large and multiple fibroids would reduce blood loss and facilitate removal of fibroids (Butori et al. 2011). More recently, it was suggested that gelatin sponge like particles reduced the risk of post-operative uterine synechia when used in post-partum context (Saiga et al. 2019).

The purpose of this study is to present our experience of per- and post-operative results following sequential PUAE using gelatin sponge-like particles prior to abdominal myomectomy.

## Materials and method

We conducted a monocentric retrospective cohort study reviewing all patients who underwent open myomectomy between January 2016 and June 2018 at Hopital Paris Saint-Joseph (HPSJ). Routinely collected, prospectively entered data from the HPSJ database were extracted and analyzed. Our institutional review board approved this study.

In this period, 121 patients underwent open myomectomy. Two patients did not give their written consent. Out of the remaining 119 patients, 16 patients had UAE 24 h prior to surgery (test group), and 103 patients had open myomectomy without UAE (control group).

All patients had ultrasound or MRI evaluation prior to the surgery.

Uterine artery embolization procedure was performed by the same interventional radiologist using a standard unilateral percutaneous transfemoral approach using Seldinger technique and a 5 -Fr sheath under local anesthesia. Superselective catheterization of the uterine artery was performed after opacification of the anterior branch of the internal iliac artery, followed by embolization using resorbable gelatin powder sheet (Curaspon®, CuraMedical, Amsterdam, The Netherlands) infused under fluoroscopic guidance until near stasis of contrast media. A post-embolization angiogram was performed to confirm successful devascularization. The procedure was then repeated for the contralateral uterine artery.

Abdominal myomectomy was performed by laparotomy by one of the 5 experienced surgeons within 24 h of the UAE.

Primary outcomes were the estimated blood loss and the difference between pre- and post-operative hemoglobin level. Secondary outcomes included the need for transfusion, surgical revision, hemostasis hysterectomy, post-operative embolization, or iron supplementation.

The statistical analysis used a propensity score (PS) adjustment process in which a propensity score is estimated for each patient and then used to construct two equal-sized cohorts with similar characteristics. Observed characteristics used to calculate the propensity score were past medical history (age, parity, past surgical myomectomy), uterine and fibroid characteristics (uterine and volume, number and size of fibroids, gross necrobiosis and hypervascularization). Pairs of similar patients were identified by matching their PS on a one-to-one basis. The quality of the matching process was tested using Fisher’s test for qualitative measures and t-student test for quantitative variables.

The statistical analysis was done assessing statistical differences in primary and secondary outcomes in the two groups using the χ^2^ test. Statistical difference was assessed at the *p*-value < 0.05 level.

## Results

During the study period, 121 patients had planned abdominal myomectomy. Two patients did not give their written consent. Out of the remaining 119 patients, 16 patients had UAE 24 h prior to the surgery, and 103 patients had abdominal myomectomy without UAE.

Table 1 shows the characteristics of the two groups. All patients had bulky uterine fibroids responsible for pelvic pain, metrorrhagia, or infertility.

**Table 1:** Patients Characteristics

Variables	Pre-op Embolization (PUAE +) *N* = 16	No embolization (PUAE -) *N* = 103	P
Age (years)	34,8	36,5	0,133
Parity	0,44	0,40	0,842
History of myomectomy (%)	25	7,8	0,056
History of Hemostasis Condition (%)	6,25	1,94	0,354
Fibroid Characteristics			
Uterine Volume (mL)	889	619	0,21
Number of Fibroids	6,19	4,24	0,096
Max Diameter (cm)	9,37	9,62	0,793
Max Volume (mL)	387	332	0,553
Hypervascularization (%)	16,7	22,2	1,000

After propensity score, the average blood loss was not significantly lower in the test group than in the control group (128 vs 192 mL, OR 1,00 [0.99;1,01], *p* = 0,73). The difference in pre- and post-operative hemoglobin level was not significant (− 1,15 g/dL in the study group vs − 1,32 g/dL in the control group, OR 0,91 [0.47;1,73], *p* = 0,79). Our bleeding composite criterion (Δ Hemoglobin level > 2 g/dL, Blood loss > 500 mL, need for per- or post-operative transfusion, post-operative embolization or surgical revision) did not show any significant difference neither in univariate analysis (9,52% in the PUAE + group vs. 14,52% in the PUAE – group, OR 0,67 [0.09;2,73], *p* = 0,73), nor after propensity score adjustment (0% in the PUAE + group vs. 25% in the PUAE – group, OR 0,91 [0.47;1,73], *p* = 0,79).

The average duration of surgery was significantly longer in in PUAE + in univariate analysis (115 min vs. 92 min, OR 1,02 [1,01;1,04], *p* = 0,01) but this result was not found after adjustment with the propensity score (114 min vs. 96 min, OR 0,91 [0.47;1,73], p = 0,79). No statistical difference was found in regard to per- and post-operative transfusion (0% in the PUAE + group vs 8,33% in the PUAE - group), surgical revision (0% in the PUAE + group vs 0,97% in the PUAE - group), post-operative embolization (0% in the PUAE + group vs 2,91% % in the PUAE - group), need for post-operative iron supplementation (6,25% in the PUAE + group vs 15,5% in the PUAE - group). Hemostasis hysterectomy did not occur in our series.

Table 2 shows the outcomes after abdominal myomectomy in the two groups.

**Table 2:** Intervention Data

	Univariate analysis				Propensity score			
Variables	PUAE + ***n*** = 16	PUAE - ***n*** = 103	OR	P	PUAE + ***n*** = 12	PUAE - ***n*** = 12	OR	P
Blood Loss (mL)	222	306	0,99 [0.99;1,01]	0,26	168	192	1,00 [0.99;1,01]	0,73
Pre-op Hemoglobin Level (g/dl)	11,9	12,1	0,91 [0.47;1,76]	0,81	12,0	10,6	1,86 [0,73;4,73]	0,21
Post-op Hemoglobin Level (g/dl)	10,8	10,8	0,99 [0.61;1,61]	0,98	11,1	10,4	1,36 [0.63;2,91]	0,48
Δ Hemoglobin Level (g/dl)	1,45	1,56	0,95 [0.64;1,41]	0,74	1,15	1,32	0,91 [0.47;1,73]	0,79
Bleeding composite criterion (%)	9,52	14,29	0,67 [0.09;2,73]	0,73	0	25	0,91 [0.47;1,73]	0,79
Operating Time (min)	115	92	1,02 [1,01;1,04]	0,01	114	96	1,02 [0.99;1,04]	0,20
Per-op Tranexamic acid injection (%)	6,25	3,88	1,80 [0.06;14,1]	0,52	8,33	0	1	1
Per- and Post-op Transfusion (%)	6,25	5,82	1,19 [0.04;8,00]	1	0	8,33	1	1
Surgical revision (%)	0	0,97	1	1	0	0	1	1
Post-op Embolization (%)	0	2,91	1	1	0	0	1	1
Iron Supplementation (%)	6,25	15,5	0,40 [0.02;2,29]	0,46	8,33	16,7	0,49 [0.01;6,99]	1

## Discussion

The present findings could not conclude that Pre-operative Uterine Artery Embolization might reduce per-operative blood loss during abdominal myomectomy.

These results are inconsistent in comparison with previous findings. In 2001, in a preliminary trial including five patients, Ngeh et al. showed the feasibility of immediate uterine artery embolization and its potential to reduce blood loss in women wishing to conserve their fertility, or simply their uterus (Ngeh et al. 2004). A descriptive study including 22 patients by Dumousset et al. in 2007 noted easier dissection, minimal intraoperative blood loss and improvement of the chances of performing conservative surgery (Dumousset et al. 2008). Üstünsöz et al. published in 2007 a retrospective cohort study including 15 patients undergoing UAE and found lower estimated blood loss and shorter operating time after UAE in comparison to a similar control group (Üstünsöz et al. 2007). In 2016, Schnapauff et al. also showed preemptive UAE facilitated a safe and uterus-preserving myomectomy, using polyvinyl alcohol particles (PVA) embolization in 21 patients (Schnapauff et al. 2018). Our two groups slightly differ in regard to history of myomectomy (25% in PUAE+ group vs. 7,6% in PUAE- group – *p* = 0,056) and the number of fibroids (6,19 in the PUAE+ group vs. 4,24 in the PUAE- group – *p* = 0,096). Patients with history of myomectomy and greater number of fibroids would be expected to have greater bleeding in regard to greater surgical complexity, and longer operating time. Yet, our results show a trend towards less bleeding and shorter operating time compared to standard myomectomy alone. With these considerations, our findings lean towards consistency with the literature.

The main limitation of our study is the lack of statistical power due to the low number of patients in the PUAE + group (*n* = 16). The propensity score adjustment process used to construct two equal-sized cohorts is a commonly used and accepted process in the literature. Fisher’s test and t-student test were realized to verify patient characteristics and imaging characteristics were not different after the matching process in respect to original cohorts. In order to improve the accuracy of our results, more patients undergoing PUAE must be included.

In the literature, estimated blood loss for abdominal myomectomy was in average 50–400 mL after PUAE (Dumousset et al. 2008; Ngeh et al. 2004; Ravina et al. 1994; Ravina et al. 1995) whereas it was estimated around 150–390 mL without PUAE (Friedman et al. 1990; Smith and Uhlir 1990). Blood transfusion varies in different studies from 2% to 7% in this context (Dubuisson et al. 1991; Friedman et al. 1988). The estimation of blood loss in our study is comparable to those found in the literature.

Various types of embolization particles have been mentioned in the literature for preemptive UAE – mainly PVA non-spherical particles, calibrated microspheres, or resorbable gelatin sponge-like particles. Resorbable sponge-like particles show a reasonably safe profile in UAE in allowing preservation of uterine trophicity and avoiding long-term ovarian ischemia, while ensuring satisfactory uterine ischemia for the surgical intervention (Dumousset et al. 2008; Butori et al. 2011; Loffroy et al. 2009). In our study, the embolization particles used were resorbable gelatin sponge-like particles, which have been used in previous studies in which PUAE was found to be useful to prevent per- and post-operative excess bleeding (Dumousset et al. 2008; Butori et al. 2011) and can therefore not be put in cause.

We did not evaluate long-term pregnancy rates in patients wishing to preserve their fertility in our cohort. Goldberg et al. noted in a review of the literature in 2006 that although UAE using PVA is a safe and effective fibroid treatment, it may cause more obstetrical complications than abdominal myomectomy, such as preterm delivery, spontaneous abortion, abnormal placentation and post-partum hemorrhage (Goldberg et al. 2002). These adverse effects tend to be overcome by the use of resorbable particles in the embolization procedure (Butori et al. 2011).

## Conclusion

Although our findings show a trend consistent with existing literature, we could not bring additional evidence showing preemptive UAE might reduce intraoperative blood loss for women with large or numerous fibroids who wish to preserve their fertility. However, in target cases such as women with history of myomectomy (possibility of adhesions, fibroids included in the parameter, isthmic fibroids), PUAE could still be a good alternative. More powerful prospective studies need to be done in order to show the benefit of this intervention.

## Data Availability

the datasets used and/or analyzed during the current study are available from the corresponding author on reasonable request.
